# Clinical and imaging features of drug-susceptible and multidrug-resistant TB in Korean adults

**DOI:** 10.5588/ijtld.23.0017

**Published:** 2023-06-01

**Authors:** S-H. Kim, J. Y. Yoo, H. S. Cho, S. R. Kim, J. Y. Cho, S. Youk, E-G. Kim, Y. M. Shin, K. H. Choe, K. M. Lee, H. Lee, B. Yang

**Affiliations:** 1 Division of Pulmonary and Critical Care Medicine, Department of Internal Medicine, Chungbuk National University College of Medicine, Cheongju, Korea; 2 Department of Radiology, Chungbuk National University College of Medicine, Cheongju, Korea; 3 Department of Internal Medicine, Chungbuk National University College of Medicine, Cheongju, Korea; 4 Department of Microbiology, Chungbuk National University College of Medicine, Cheongju, Korea; 5 Department of Biochemistry, Chungbuk National University College of Medicine, Cheongju, Korea; 6 Division of Pulmonary Medicine and Allergy, Department of Internal Medicine, Hanyang University College of Medicine, Seoul, Republic of Korea

Dear Editor,

TB is the leading cause of mortality due to an infectious disease. The treatment success rate of drug-susceptible TB (DS-TB) is approximately 86%, but that of multidrug-resistant TB (MDR-TB) is approximately 59%.^1^ Early detection of MDR-TB is essential for public health infection control and to allow for prompt and effective treatment. Drug susceptibility testing (DST) is essential for diagnosing resistant TB, including MDR-TB, and selecting therapeutic agents. However, it takes approximately 80 days to confirm the phenotypic DST result, and there is a risk of treatment delay and drug resistance acquisition.^2^ Therefore, when diagnosing TB, clinical and radiological characteristics that can quickly identify potential drug resistance are important. Previous studies have reported that multiple cavities, bronchiectasis and bilateral lung involvement are more common in cases of MDR-TB than with DS-TB.^3^–^5^ A positive sputum acid-fast bacilli (AFB) smear and a history of TB are known risk factors for MDR-TB.^6^ However, studies on comprehensive clinical and radiological differences between MDR-TB and DS-TB are lacking. We therefore investigated and compared the clinical features and radiological findings of MDR-TB and DS-TB.

We reviewed the medical records of 635 consecutive patients diagnosed with culture-proven pulmonary TB between January 2014 and December 2021. Of these 635 patients, we excluded those with drug-resistant TB (except MDR-TB) or extensively drug-resistant TB (XDR-TB) (*n* = 52), those without DST results (*n* = 14) and those who did not undergo chest computed tomography (CT) (*n* = 42). A total of 527 patients were included and demographic and comorbidity data were collected through a retrospective review of the medical records. Two radiologists reviewed all chest CT images in a blinded manner, and decisions were reached by consensus. Sputum AFB smears and cultures were prepared using standard methods.^7^ Conventional DST was performed using the absolute concentration method and Löwenstein-Jensen media at the Korean Institute of Tuberculosis (Osong, Republic of Korea).^8^ We compared continuous variables using the Mann–Whitney *U*-test, and categorical variables using Pearson’s χ^2^ test or Fisher’s exact test. Additionally, multivariable logistic regression analysis was used to analyse factors related to MDR-TB into radiological and clinical factors. Factors included age, sex, body mass index, previous history of TB, comorbidities and AFB smear results. The study was approved by the Institutional Review Board of Chungbuk National University Hospital, Cheongju, Republic of Korea (IRB No. 2022-08-028).

**Figure 1:**
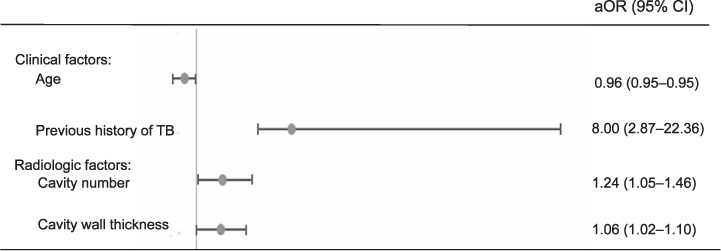
Multivariate logistic regression analysis of the clinical factors and radiologic factors associated with the development of multidrug-resistant TB. aOR = adjusted odds ratio; CI = confidence interval.

**Figure 2:**
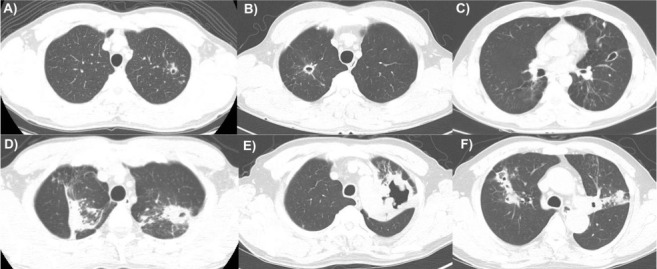
Characteristic CT findings of MDR-TB and DS-TB. A–C) DS-TB in a 52-year-old man. Chest CT axial images with lung window settings showed several cavities in both lungs. The average wall thickness of the cavities is approximately 6 mm. D–F) MDR-TB in a 62-year-old man. Chest CT axial images with a lung window setting showed six or more cavities in both lungs. The walls were relatively thick (>11 mm). CT = computed tomography; MDR-TB = multidrug-resistant TB; DS-TB = drug-susceptible TB.

The baseline characteristics of the 527 patients with pulmonary TB are shown in Supplementary Table S1. Among the 527 patients, 488 (92.6%) had DS-TB, and 39 (7.4%) had MDR-TB. The patients with MDR-TB were younger than those with DS-TB (median age: 48 years vs. 66 years; *P* < 0.001) and more frequently had a history of TB (51.3% vs. 10.0%, *P* < 0.001). The chest CT findings of all patients are shown in Supplementary Table S2. The number of cavities was higher in patients with MDR-TB than in those with DS-TB (3, IQR 1–6 vs. 2, IQR 1–3; *P*=0.024). There was no significant difference in cavity size between the two groups, but the cavity wall was thicker in patients with MDR-TB than in those with DS-TB (15 mm, IQR 11–25 vs. 11 mm, IQR 6–17; *P* = 0.010). Multivariable analysis showed that age (adjusted odds ratio [aOR] 0.96, 95% confidence interval [CI] 0.95–0.98) and previous history of TB (aOR 8.00, 95% CI 2.87–22.36) were significantly associated with MDR-TB. Multivariable analysis showed that the number of cavities (aOR 1.24, 95% CI 1.05–1.46) and cavitary wall thickness (aOR 1.06, 95% CI 1.02–1.10) were significantly associated with MDR-TB (Figure 1). The characteristic findings of chest CT in MDR-TB and DS-TB is shown in Figure 2.

Our study shows that younger age and previous history of TB were significantly associated with MDR-TB, and chest CT scans revealed that more cavities and thicker cavity wall thickness were significantly associated with MDR-TB. Previous studies have also reported that younger age is associated with MDR-TB.^4^,^9^ The higher incidence of MDR-TB in younger patients is attributable to higher social activity and increased exposure to contacts with MDR-TB. The possibility of MDR-TB should therefore be considered when diagnosing young patients with TB. Another clinical factor associated with MDR-TB is previous treatment. According to a recent report,^10^ MDR-TB is transmitted from person to person and is not necessarily acquired in countries with high incidence of TB. However, other studies (including ours), have reported that previous treatment for TB is strongly associated with MDR-TB.^6^,^11^ Drug resistance may have emerged with previous anti-TB chemotherapy, but further research is needed to clarify this.

Our study revealed differences in the chest imaging of patients with DS-TB and MDR-TB. Among the several imaging patterns of chest CT scans, patients with MDR-TB and DS-TB showed significant differences in the number of cavities and cavity wall thickness. First, as reported in previous studies,^4^–^6^ patients with MDR-TB had significantly more cavities than DS-TB patients. A possible reason for this is that bacterial replication is active because of the high oxygen concentration in the cavity,^12^ and such rapid replication may increase the frequency of mutations during the replication process and result in drug resistance.^13^ A second notable finding was that the cavity wall of patients with MDR-TB was thicker than that of patients with DS-TB. Another study reported similar findings, but the wall thickness was not measured.^14^ To overcome this limitation, we quantitively measured wall thickness of cavities and observed that there was a significant difference in cavity thickness between patients with MDR-TB and DS-TB. The cavity wall may act as a barrier against the immune defense system due to its limited vascularisation. Because it is difficult for immune cells to penetrate thick-walled cavities, increased thickness might contribute to the continuous growth of bacteria and high bacterial loads, which may increase the risk of spontaneous genetic mutations.^12^

There are some limitations to our study. First, the number of subjects with MDR-TB was relatively small and future studies with larger cases numbers of MDR-TB are needed to confirm our findings. Second, although our findings are interesting, it is unlikely they can be operationalised or implemented pro-grammatically to improve drug-resistant TB screening/diagnostic algorithm. However, we believe our study findings provide interesting basic science knowledge for the field of chest imaging of cavities and the development of MDR-TB.

In conclusion, our study showed that young age, history of TB, number of cavities and cavity wall thickness were all significantly associated with MDR-TB.
